# Underlying Medical Conditions and Hospitalization for Pandemic (H1N1) 2009, Japan

**DOI:** 10.3201/eid1610.091755

**Published:** 2010-10

**Authors:** Taro Tomizuka, Yoshihiro Takayama, Tokuaki Shobayashi, Yasumasa Fukushima, Yasuhiro Suzuki

**Affiliations:** Author affiliations: National Institute of Public Health, Saitama, Japan (T. Tomizuka, Y. Suzuki);; Ministry of Health, Labour and Welfare, Tokyo, Japan (T. Tomizuka, Y. Takayama, T. Shobayashi, Y. Fukushima, Y. Suzuki);; Tokyo Medical and Dental University, Tokyo (T. Tomizuka)

**Keywords:** influenza A virus, risk, comorbidity, age-specific incidence, hospitalization, viruses, influenza, pandemic (H1N1) 2009, Japan, letter

**To the Editor:** Early epidemiologic reports suggested that infection with pandemic (H1N1) 2009 virus most commonly occurred in teenagers and young adults (*1*). Although this infection appears to have a mild clinical course and the mortality rate has been relatively low in Japan (1.6 deaths/1 million population) (*2*), the reported number of patients with severe (requiring intubation or admission to an intensive care unit) cases of this disease has been increasing (*2*). The difference in severity of pandemic (H1N1) 2009 infection may be attributed to differences in underlying medical conditions or to age-related differences in susceptibility. To explore these differences, we investigated the incidence of laboratory-confirmed cases of pandemic (H1N1) 2009 virus infections resulting in hospitalization in Japan and the patients’ age-specific risks for hospitalization associated with underlying medical conditions.

During the outbreak in Japan, hospitals, local public health centers, and local governments were required to report hospitalizations associated with pandemic (H1N1) 2009 virus to the Ministry of Health, Labour and Welfare in Japan. Infection was confirmed by PCR at local public health centers. The data for hospitalized patients were integrated through a nationwide surveillance system, the interim National Epidemiologic Surveillance of Infectious Diseases.

We collected data on patients who were hospitalized in Japan for pandemic (H1N1) 2009 virus infection from July 28 through December 14, 2009. The number of new cases (incidence) increased in the middle of August and peaked at the end of November (*2*). We excluded from analysis those patients who had been hospitalized for the purpose of infection containment. Medical conditions were defined as diseases that are risk factors for severe illness associated with seasonal influenza: chronic respiratory diseases, chronic cardiovascular diseases, chronic renal diseases, chronic liver diseases, neurologic diseases, hematologic diseases, diabetes, and immunosuppression caused by treatments or illnesses (including malignant neoplasm) and chronic childhood diseases (*3**,**4*). Data on the prevalence of the diseases among the population were obtained from the 2005 National Patient Survey and from population estimates obtained by the Ministry of Internal Affairs and Communications in May 2009.

During the surveillance period, 12,702 laboratory-confirmed cases for which the patients required hospitalization were reported; 110 of these patients died (Table). Hospitalization incidence was 10.0 admissions/100,000 persons (overall incidence of hospitalization in the general population in 2005 was 1,462.8 admissions/100,000 persons). Median age of hospitalized patients was 7 years (interquartile range 5–11 years). Of the 10,721 patients for whom type of care was known, 680 (6.4%) were admitted to an intensive care unit. Among 486 hospitalized women 15–44 years of age, only 42 (8.6%) were pregnant; this percentage is small compared with results of studies conducted in other countries (*5**–**7*). None of the pregnant women were in critical condition and none died of pandemic (H1N1) 2009 infection during the surveillance period. The overall lower prevalence of pregnant women in Japan (≈0.67% of the total population) compared with that in other countries (*8*) might account for the low number of pregnant women hospitalized for pandemic (H1N1) 2009 virus in Japan.

**Table:** Age-specific incidence and risk for hospitalization with laboratory-confirmed pandemic (H1N1) 2009 virus infection, Japan, July 28–December 14, 2009*

Patient characteristic	Underlying medical conditions†			No underlying medical conditions‡		RR	AR
	No. (%) patients	Incidence/100,000§		No. (%) patients	Incidence/100,000§		
Age group, y							
0–4	648 (22.1)	195.2		2,285 (77.9)	45.2	4.3	150.0
5–9	1,848 (32.6)	814.1		3,822 (67.4)	69.4	11.7	744.7
10–14	763 (35.2)	573.7		1,406 (64.8)	24.0	23.9	549.6
15–19	173 (38.9)	219.0		272 (61.1)	4.5	48.4	214.5
20–29	110 (43.8)	53.9		141 (56.2)	1.0	54.9	52.9
30–39	105 (49.8)	28.5		106 (50.2)	0.6	48.4	27.9
40–49	125 (61.6)	21.6		78 (38.4)	0.5	43.5	21.1
50–59	176 (76.9)	12.8		53 (23.1)	0.3	38.3	12.5
60–69	148 (77.5)	6.7		43 (22.5)	0.3	24.0	6.5
>70	324 (81.0)	8.2		76 (19.0)	0.5	17.9	7.8
Gender*¶*							
M	2,857 (35.3)	NA		5,236 (64.7)	NA		
F	1,563 (33.9)	NA		3,046 (66.1)	NA		
*RR, relative risk for hospitalization; AR, attributable risk for hospitalization; NA, not available. Medical conditions defined as chronic respiratory diseases, chronic cardiovascular diseases, chronic renal diseases, chronic liver diseases, neurologic diseases, hematologic diseases, diabetes, and immunosuppression (including malignant neoplasm) and chronic childhood diseases. †n = 4,420 (43.8%); 83 patients died. ‡n = 8,282 (65.2%); 27 patients died. §Incidence = no. case-patients with pandemic (H1N1) 2009 virus infection and a particular medical condition/total population having the same medical condition (e.g., population 0–4 years of age with medical condition). ¶p value for difference in gender was 0.11, obtained by χ^2^ test (2-sided) for testing the null hypothesis.							

Of all 12,702 patients, 4,420 (34.8%) had underlying medical conditions. In terms of age, 1,848 (32.6%) of 5,670 patients 5–9 years of age and 324 (81.0%) of 400 patients >70 years of age had underlying medical conditions. For those with underlying medical conditions, incidence of hospitalization was relatively higher for children than for adults; rates for hospitalized children were 814.1, 573.7, and 219.0 per 100,000 persons for those 5–9, 10–14, and 15–19 years of age, respectively; whereas, the rate for hospitalized adults 60–69 years of age was 6.7/100,000 persons. In all age categories, the incidence of hospitalization for pandemic (H1N1) 2009 virus infection was notably higher for patients with underlying medical conditions than for those without. The attributable risk for medical conditions associated with hospitalization for pandemic (H1N1) 2009 virus infection was highest among patients 5–9 and 10–14 years of age (744.7 and 549.6, respectively), whereas the risk was lowest (6.5) among patients 60–69 years of age. The relative risk associated with underlying medical conditions was comparatively higher for patients in age groups 15–19, 20–29, and 30–39 years (48.4, 54.9, and 48.4, respectively) than among those >70 years of age (17.9). In contrast to seasonal influenza (*4*), persons <20 years of age had higher risk for hospitalization for pandemic (H1N1) 2009 than did older age groups.

Considering our findings regarding the attributable risk for hospitalization, interventions that aim to control pandemic (H1N1) 2009 virus infection among children, especially those with underlying medical conditions, can be considered key for minimizing the strain (financial, staffing, space) on the healthcare system. Our findings justify prioritizing the treatment of children and young adults by vaccination and early prescription of antiviral drugs.
